# Comparative Value of Four Measures of Retention in Expert Care in Predicting Clinical Outcomes and Health Care Utilization in HIV Patients

**DOI:** 10.1371/journal.pone.0120953

**Published:** 2015-03-20

**Authors:** Kelly R. Reveles, Timothy R. Juday, Matthew J. Labreche, Eric M. Mortensen, Jim M. Koeller, Daniel Seekins, Christine U. Oramasionwu, Mary Bollinger, Laurel A. Copeland, Xavier Jones, Christopher R. Frei

**Affiliations:** 1 The University of Texas at Austin, Austin, Texas, United States of America; and The University of Texas Health Science Center at San Antonio, San Antonio, Texas, United States of America; 2 Bristol-Myers Squibb Company, Plainsboro, New Jersey, United States of America; 3 The Johns Hopkins Hospital, Baltimore, Maryland, United States of America; 4 VA North Texas Health Care System and The University of Texas Southwestern Medical Center, Dallas, Texas, United States of America; 5 University of North Carolina at Chapel Hill, Chapel Hill, North Carolina, United States of America; 6 South Texas Veterans Health Care System, San Antonio, Texas, United States of America; 7 Central Texas Veterans Health Care System and Scott & White Healthcare, Center for Applied Health Research, Temple, Texas, United States of America; Azienda ospedaliero-universitaria di Perugia, ITALY

## Abstract

This study compared the ability of four measures of patient retention in HIV expert care to predict clinical outcomes. This retrospective study examined Veterans Health Administration (VHA) beneficiaries with HIV (ICD-9-CM codes 042 or V08) receiving expert care (defined as HIV-1 RNA viral load and CD4 cell count tests occurring within one week of each other) at VHA facilities from October 1, 2006, to September 30, 2008. Patients were ≥18 years old and continuous VHA users for at least 24 months after entry into expert care. Retention measures included: Annual Appointments (≥2 appointments annually at least 60 days apart), Missed Appointments (missed ≥25% of appointments), Infrequent Appointments (>6 months without an appointment), and Missed or Infrequent Appointments (missed ≥25% of appointments or >6 months without an appointment). Multivariable nominal logistic regression models were used to determine associations between retention measures and outcomes. Overall, 8,845 patients met study criteria. At baseline, 64% of patients were virologically suppressed and 37% had a CD4 cell count >500 cells/mm^3^. At 24 months, 82% were virologically suppressed and 46% had a CD4 cell count >500 cells/mm^3^. During follow-up, 13% progressed to AIDS, 48% visited the emergency department (ED), 28% were hospitalized, and 0.3% died. All four retention measures were associated with virologic suppression and antiretroviral therapy initiation at 24 months follow-up. Annual Appointments correlated positively with CD4 cell count >500 cells/mm^3^. Missed Appointments was predictive of all primary and secondary outcomes, including CD4 cell count ≤500 cells/mm^3^, progression to AIDS, ED visit, and hospitalization. Missed Appointments was the only measure to predict all primary and secondary outcomes. This finding could be useful to health care providers and public health organizations as they seek ways to optimize the health of HIV patients.

## Introduction

According to the Centers for Disease Control and Prevention (CDC), nearly 900,000 people in the United States (U.S.) were living with diagnosed human immunodeficiency virus (HIV) infection by the end of 2010. The estimated incidence of HIV in the U.S. during the same year was 49,300 infections [1]. The advent of highly active antiretroviral therapy (HAART) in the mid-1990s has greatly increased the estimated life expectancy of patients with HIV following diagnosis, from 5.6 years in 1993 to 24.2 years in 2006 [2,3].

HIV expert care is defined as HIV-1 RNA viral load and CD4 cell count tests occurring within one week of each other. Retention in expert care is a critical component in optimizing the health outcomes of HIV patients, by closely monitoring markers of HIV severity and progression. Research has demonstrated that retention in HIV care is associated with improvement in CD4 cell count, reduction in HIV viral load, reduction in opportunistic infections, and improved survival [4–9]. Retention might also improve acceptance of HIV status and promote social support through intrapersonal relationships, each of which might also be important for improved health outcomes [10].

It is estimated that only about half of all HIV patients in the U.S. routinely receive HIV-focused care [11]. Prior studies have demonstrated that 34–60% of HIV patients miss appointments with their HIV care provider and 39% of patients do not have regular follow-up [6,8,9]. In low- to middle-income countries, studies have reported substantial loss of patients, from HIV testing to treatment initiation [12]. These data suggest that linkage and retention in HIV expert care are significant barriers to optimizing health outcomes among patients with HIV.

Several organizations, including the National Committee for Quality Assurance (NCQA), National Quality Forum (NQF), and the Human Resources and Services Administration HIV/AIDS Bureau (HRSA HAB) include retention in HIV expert care as a marker of quality care; however, there is no standard definition for measuring retention. A number of different measures have been used in published studies in an attempt to quantify patient retention in HIV expert care [13–15]. Patient retention is most commonly reported as: 1) appointments missed, 2) medical visits at regularly defined intervals, or 3) a combination of these two measures [14]. The NCQA defined retention in HIV expert care as patients being seen by their provider at least twice annually at least 60 days apart [13]. Similarly, the HRSA HAB defined retention in HIV expert care as at least two appointments annually at least 90 days apart. In addition, retention measures vary widely in low- to middle-income countries. These measures, which often include various amounts of elapsed time between visits, have made it difficult to make comparisons across programs [16,17].

Few studies have compared the predictive value of these retention in care measures among HIV patients. The aim of this study was to assess which measure of retention in HIV expert care was most predictive of patient outcomes in a national cohort of U.S. Veterans Health Administration (VHA) patients. We hypothesized that each of the studied retention measures would be predictive of patient outcomes and health care utilization, and that certain measures would have a greater effect on outcomes.

## Methods

### Study design and data source

This was a retrospective cohort study of patients initiating HIV expert care at any U.S. VHA facility between October 1, 2006 and September 30, 2008 with an additional 24 months of follow-up. The VHA is the largest integrated health care system in the U.S. and includes more than 150 hospitals and 850 clinics. In 2006, there were approximately 23 million VHA enrollees in the U.S. [18] Data for this study were obtained from the VHA electronic medical record system which includes administrative, clinical, laboratory, and pharmacy data repositories. Four national VA databases were used: the VA Medical SAS Datasets (both inpatient and outpatient), the VA Vital Status File, the VA Decision Support System Datasets, and the VHA Annual Enrollment Files. The VA Medical Statistical Analysis System (SAS) Inpatient Dataset includes patient demographics, diagnoses, procedures, and discharge status. The VA Vital Status File contains date of birth, date of death, and gender for each veteran. The VA DSS integrates data from clinical and financial systems to create National Data Extracts (NDEs). NDEs for clinical data include laboratory results and both inpatient and outpatient pharmacy records. Finally, the VHA Annual Enrollment Files contain eligibility information used to create priority groups. The University of Texas Health Science Center at San Antonio Institutional Review Board (San Antonio, TX) and the South Texas Veterans Health Care System Research and Development Committee (San Antonio, TX) approved this study. This study design did not require patient informed consent to view medical records. Patient medical information was anonymized and de-identified prior to analysis.

### Patient eligibility

The study population included HIV patients managed at VHA health care facilities between October 1, 2006 and September 30, 2010. Patients were included in the study sample if: (1) they entered into expert care (defined as HIV-1 RNA viral load and CD4 cell count tests occurring within one week of each other) between October 1, 2006 and September 30, 2008; (2) were diagnosed with HIV/acquired immune deficiency syndrome (AIDS) (defined as an ICD-9-CM code of 042 or V08 in any position on at least one outpatient or inpatient medical encounter) within six months of entry into expert care (between April 1, 2006 and March 31, 2009); (3) were at least 18 years old on the date of entry into expert care; (4) had HIV-1 RNA viral load and CD4 cell count tests occurring within one week of each other at 24 ± 3 months after the date of entry into expert care; and (5) were a continuous VHA user for at least 24 months after entry into expert care. The latter two inclusion criteria were chosen to ensure that the patient was still receiving HIV expert care in the two years following study entry and to allow adequate time to assess patient outcomes and healthcare utilization. A 24-month follow-up period has been used in prior studies that evaluated the association of retention measures and patient outcomes [19,20].

### Definitions

Patient demographics included: age, sex, race, ethnicity, and marital status. Age was calculated as the date at study inclusion minus the date of birth plus one day. Sex, race, ethnicity, and marital status were defined as the most common reporting of these demographics, as indicated by the medical records, over the study period. Retention in HIV expert care was defined using four variables: Annual Appointments, Missed Appointments, Infrequent Appointments, and Missed or Infrequent Appointments. Annual Appointments was defined using the NCQA definition for retention: ≥2 appointments annually at least 60 days apart. At least 2 appointments were expected per year [21]. Missed Appointments was defined as missing at least 25% of appointments. This proportion was derived from the number of appointments for which the veteran had a “no show” divided by the total number of "no shows" plus the number of actual appointments. “No shows” are those appointments that are missed without prior notification or rescheduling. This methodology has been used in prior studies that assessed retention in HIV expert care [8,22].

Missed Appointments was further divided into appointments missed at any clinic and those missed at infectious diseases (ID) clinics. Infrequent Appointments represents gaps in care and was defined as >6 months without an appointment. Finally, Missed or Infrequent Appointments was determined by combining two of the variables (missed ≥25% of appointments or >6 months without an appointment).

Covariates included demographic measures, treatment facility, and VHA Priority status, a categorization from 1–8 describing why the veteran was eligible for VHA care. Priority 1 veterans are 50%-100% disabled by a military service-related condition and have no co-pays for care or prescription medications. Priority 2–6 veterans have medication co-pays. Priority 7–8 veterans have agreed to pay co-pays for care and medications and are ineligible under Priorities 1–6. Priority status correlates with both higher illness severity and lower socioeconomic status, and has been validated with VHA administrative data [23–26].

### Patient outcomes

The primary outcomes were: HIV-1 RNA viral load and CD4 cell count at 24 months post-baseline. HIV-1 RNA viral load was stratified into two groups: <500 copies/mL versus ≥500 copies/mL [21]. This HIV-1 RNA viral load threshold was chosen because all sites employed an assay with at least a minimum detection value of 500 copies/mL throughout the study period [27]. CD4 cell count was stratified into four groups: CD4 cell count <200, 200–349, 350–500, or >500 cells/mm^3^. These are the cutoff values for initiation of HAART and opportunistic infection prophylaxis according to the current guidelines [21,28]. Furthermore, these values are commonly used to determine the stage of HIV illness [29,30].

Secondary analyses assessed five patient outcome measures over 24 months of follow-up: (1) incidence of AIDS-defining conditions, (2) all-cause emergency department (ED) visits, (3) all-cause hospitalizations, and (4) initiation of antiretroviral therapy (ART). ART initiation was defined as the receipt of any antiretroviral medication used to treat HIV [31]. Progression to AIDS was defined as a CD4 cell count <200 cells/mm^3^ and/or evidence of one or more of the CDC AIDS-defining conditions [32]. Patients already on ART were excluded from the ART outcome analysis. Likewise, patients who already had a diagnosis of AIDS at baseline were excluded from the AIDS outcome analysis. We had originally planned to study patient mortality as an additional outcome in our secondary analyses; however, there were insufficient numbers of deaths to assess the predictive value of the measures for death.

### Data and statistical analysis

SAS 9.2 and JMP 9.0 (SAS Corp., Cary, NC) were used for all analyses. Continuous variables are presented as means and standard deviations (SD). Categorical variables are presented as the number and percentage of subjects in each category.

We utilized logistic regression models to evaluate the association of retention in care measures with patient outcomes. Categorical covariates included: sex, race, marital status, VHA priority group, HIV-1 viral load (dichotomized to <500 copies/mL and ≥500 copies/mL), CD4 cell count (dichotomized to ≤500 cells/mm^3^ and >500 cells/mm^3^), and treatment facility; whereas, patient age was a continuous variable. Charlson comorbidities were collected using ICD-9-CM codes and were dichotomized (yes/no). We also calculated the Charlson comorbidity score as a continuous variable according to Deyo et al. [33]. All covariates were collected at baseline and were tested for multicollinearity using the Spearman rank correlation test. Each of these covariates was chosen because they have been previously shown to be associated with clinical outcomes among patients with HIV [34–37]. Missing data were excluded from analyses. Results are presented as adjusted odds ratios (OR) and p-values. The retention measure most predictive of patient outcomes was identified by comparing the ORs of the associations within the regression models.

## Results

### Baseline characteristics

Overall, 8,845 patients met study inclusion criteria. Table 1 describes the patients’ baseline characteristics. Patients had a mean age of 52 ± 10 years. Patients were predominately male (97%) and of Black race (51%). The mean Charlson comorbidity score was 6.6 ± 2.1 (the score weights HIV as 2 points and AIDS as 6 points). Common comorbidities included: hypertension (36%), dyslipidemia (31%), and diabetes (14%). Of the baseline characteristics included, no set of variables had a correlation coefficient greater than 0.2, indicating low multicollinearity among study variables. The majority of patients (74%) were receiving ART at baseline. Also at baseline, 64% of patients were virologically suppressed and 37% had a CD4 cell count >500 cells/mm^3^. AIDS (26%) was present in some of our patients at baseline. Approximately 7% of patients also had an AIDS-defining condition at baseline. Patients with Missed Appointments differed from those without Missed Appointments with regard to baseline demographics, CD4 cell count, HIV-1 RNA viral load, and several comorbidities.

**Table 1:** Patient baseline characteristics.

Variables	Subcategory	Overall (n = 8,845)	Missed Appointments (n = 5,786)	No Missed Appointments (n = 3,059)	P-value
**Age (yrs), mean ± SD**		52 ± 10	51 ± 9	54 ± 10	<0.0001
**Male, n (%)**		8,608 (97%)	5,597 (97%)	3,011 (98%)	<0.0001
**Race & ethnicity, n (%)**	Black, non-Hispanic	4,466 (51%)	3,314 (57%)	1,152 (38%)	<0.0001
	White, non-Hispanic	3,010 (34%)	1,624 (28%)	1,386 (45%)	<0.0001
	Hispanic	496 (6%)	364 (6%)	132 (4%)	<0.0001
	Other, non-Hispanic	484 (5%)	260 (4%)	224 (7%)	<0.0001
**Married, n (%)**		1,225 (14%)	801 (14%)	424 (14%)	0.9825
**HIV-1 RNA viral load, n (%)**	Suppressed (<500 copies/mL)	5,612 (64%)	3,311 (57%)	2,301 (75%)	<0.0001
	Non-suppressed (≥ 500 copies/mL)	3,233 (37%)	2,475 (43%)	758 (25%)	<0.0001
**CD4 cell count, n (%)**	<200 cells/mm^3^	1,937 (22%)	1,376 (24%)	561 (18%)	<0.0001
	200–349 cells/mm^3^	1,786 (20%)	1,202 (21%)	584 (19%)	0.0607
	350–500 cells/mm^3^	1,884 (21%)	1,218 (21%)	666 (22%)	0.4308
	>500 cells/mm^3^	3,238 (37%)	1,990 (34%)	1,248 (41%)	<0.0001
**VHA Priority Status, n (%)**	Priority 1	1,660 (19%)	1,215 (21%)	445 (14%)	<0.0001
	Priority 2–6	6,356 (72%)	4,259 (74%)	2,160 (71%)	<0.0001
	Priority 7–8	749 (8%)	302 (5%)	447 (15%)	<0.0001
**Charlson score, mean ± SD**		6.6 ± 2.1	6.7 ± 2.2	6.6 ± 1.9	0.0002
**Charlson variables, n (%)**	Congestive heart failure	164 (2%)	119 (2%)	45 (1%)	0.0521
	Chronic obstructive pulmonary disease	925 (11%)	662 (11%)	263 (9%)	<0.0001
	Cerebrovascular disease	239 (3%)	170 (3%)	69 (2%)	0.0597
	Diabetes with complications	176 (2%)	127 (2%)	49 (2%)	0.0574
	Diabetes without complications	1,098 (12%)	760 (13%)	338 (11%)	0.0047
	Hemi/paraplegia	33 (0.4%)	26 (0.4%)	7 (0.2%)	0.1056
	HIV (as defined by Charlson)	2,173 (25%)	1,512 (26%)	661 (22%)	<0.0001
	AIDS (as defined by Charlson)	8,134 (92%)	5,285 (91%)	2,849 (93%)	0.0032
	Liver (mild disease)	27 (0.3%)	18 (0.3%)	9 (0.3%)	0.8911
	Liver (mod/severe disease; cirrhosis)	115 (1%)	75 (1%)	40 (1%)	0.9641
	Cancer	536 (6%)	341 (6%)	195 (6%)	0.3671
	Leukemia	11 (0.1%)	8 (0.1%)	3 (0.1%)	0.6099
	Metastatic cancer	27 (0.3%)	15 (0.3%)	12 (0.4%)	0.2807
	Myocardial infarction	97 (1%)	64 (1%)	33 (1%)	0.9065
	Peptic ulcer disease	73 (0.8%)	51 (0.9%)	22 (0.7%)	0.4224
	Peripheral vascular disease	159 (2%)	112 (2%)	47 (2%)	0.1789
	Renal disease	463 (5%)	313 (5%)	150 (5%)	0.3095
	Dyslipidemia	2,772 (31%)	1,506 (26%)	1,266 (41%)	<0.0001
	Hypertension	3,190 (36%)	2,109 (36%)	1,081 (35%)	0.3004
**Antiretroviral therapy, n (%)**		6,651 (74%)	4,043 (70%)	2,518 (82%)	<0.0001
**AIDS, n (%)**		2,268 (26%)	1,603 (28%)	665 (22%)	<0.0001
**AIDS-defining condition, n (%)**		610 (7%)	428 (7%)	182 (6%)	0.0106

AIDS = acquired immune deficiency syndrome; HIV = human immunodeficiency syndrome; SD = standard deviation

Note: Priority 1 veterans are 50%–100% disabled by a military service-related condition and have no co-pays for care or prescription medications; Priority 2–6 veterans have medication co-pays; Priority 7–8 veterans have agreed to pay co-pays for care and medications and are ineligible under Priorities 1–6.

Note: Charlson score defines HIV/AIDS using ICD-9-CM code 042 and does not include asymptomatic HIV infection (ICD-9-CM code V08)

### Retention measures

Retention measures at 24 months are presented in Table 2. Overall, 3,567 patients (40%) had Annual Appointments, 5,786 (65%) had Missed Appointments, 4,958 (56%) had Infrequent Appointments, and 7,166 (81%) had Missed or Infrequent Appointments. When the analyses were limited to Missed Appointments for infectious diseases clinics, the numbers were 36% for Missed Appointments and 68% for Missed or Infrequent Appointments.

**Table 2:** Retention measures at 24 months.

Variables, n (%)	Cohort (n = 8,845)
**Annual Appointments (≥2 appointments annually at least 60 days apart)**	3,567 (40%)
**Missed Appointments (missed ≥25% of appointments)**	5,786 (65%)
**Infrequent Appointments (>6 months without an appointment)**	4,958 (56%)
**Missed or Infrequent Appointments (missed ≥25% of appointments or >6 months without an appointment)**	7,166 (81%)

### Patient outcomes

The proportion of patients with CD4 cell counts >500 cells/mm^3^ increased from 37% at baseline to 46% at 24 months (*p*<0.0001) (Tables 1 and 3). The proportion of patients with virologic suppression also increased from baseline (64%) to follow-up (82%) (*p*<0.0001). During follow-up, 13% of patients progressed to AIDS and <1% died. Herpes simplex-associated chronic ulcers (4%) and pneumonia (3%) were the most common AIDS-defining conditions. Nearly half (48%) of all patients had at least one ED visit over the study period and 28% were hospitalized.

**Table 3:** Patient outcomes at 24 months.

Variables	Subcategory	Cohort (n = 8,845)
**HIV-1 RNA viral load, n (%)**	Suppressed (<500 copies/mL)	7,235 (82%)
	Not suppressed (≥ 500 copies/mL)	1,610 (18%)
**CD4 cell count, n (%)**	<200 cells/mm^3^	1,101 (12%)
	200–349 cells/mm^3^	1,680 (19%)
	350–500 cells/mm^3^	2,015 (23%)
	>500 cells/mm^3^	4,049 (46%)
Initiation of ART, n (%)*		1,581/2,284 (69%)
Incidence of AIDS, n (%)*		853/6,577 (13%)
**ED visit, n (%)**		4,249 (48%)
**ED visits, mean ± SD**		1.5 ± 3.0
**Hospitalizations, n (%)**		2,494 (28%)
**Hospitalizations, mean ± SD**		0.8 ± 1.9
**Death, n (%)**		26 (0.3%)

*Patients who had these characteristics at baseline were excluded from these results.

ED = emergency department; ART = antiretroviral therapy; AIDS = acquired immune deficiency syndrome; SD = standard deviation

Each of the four retention measures was associated with one or more patient outcomes. In unadjusted logistic regression models, all four retention measures were associated with virologic response. Additionally, Missed Appointments and Appointments Missed or Infrequent were negatively associated with CD4 cell count >500 cells/mm^3^ and positively associated with ART initiation, progression to AIDS, hospital admission, and ED visits.

The results of our multivariable, adjusted logistic regression models are presented in Table 4. All four retention measures were associated with virologic suppression at 24 months. Annual Appointments were positively associated with virologic response, while the remaining three retention measures were negatively associated with virologic response. Similarly, all four retention measures predicted ART initiation at follow-up. Annual Appointments also predicted CD4 cell count >500 cells/mm^3^. Importantly, Missed Appointments was significantly associated with all clinical outcomes, including CD4 cell count >500 cells/mm^3^ (negative predictor), progression to AIDS, ED visit, and hospitalization. Finally, Missed or Infrequent Appointments was also associated with ED visits and hospital admission.

**Table 4:** Multivariable nominal logistic regression models for patient outcomes at 24 months: OR (p-value), n = 8,845^a^ ^,^ ^b^ ^,^ ^c^.

Retention Measures, adjusted OR (95% CI)	Viral Load <500 copies/mL	CD4 Count >500 cells/mm^3^	ART	AIDS	All-Cause ED Visit	All-Cause Hospitalization
Annual Appointments	**1.40 (<0.0001)**	**1.32 (0.0003)**	**1.74 (<0.0001)**	0.88 (0.1414)	0.96 (0.3716)	1.04 (0.5279)
Missed Appointments	**0.66 (<0.0001)**	**0.80 (0.0038)**	**0.69 (0.0098)**	**1.30 (0.0028)**	**2.42 (<0.0001)**	**2.63 (<0.0001)**
Infrequent Appointments	**0.61 (<0.0001)**	0.97 (0.6948)	**0.72 (0.0046)**	0.95 (0.5492)	0.95 (0.2713)	0.92 (0.1441)
Missed or Infrequent Appointments	**0.54 (<0.0001)**	0.84 (0.0572)	**0.55 (0.0014)**	1.15 (0.1945)	**1.90 (<0.0001)**	**1.90 (<0.0001)**

^a^Covariates were: patient age, sex, race, marital status, priority group, baseline viral load, baseline CD4 count, Charlson comorbidity score, and treatment facility.

^b^Patients with outcome of interest at baseline were excluded from these analyses

^c^Bold indicates statistical significance

AIDS = progression to acquired immune deficiency syndrome; ED = emergency department; ART = antiretroviral therapy initiation

## Discussion

This is one of the first studies to compare clinical outcomes in patients using different measures for retention in HIV expert care in the U.S., a high-income country. Our study provides evidence that each of the four retention measures in this study has clinical value; however, Missed Appointments was the only measure to predict all of our primary and secondary outcomes. This is important because the definition chosen to measure retention in HIV expert care might have implications for patient health outcomes, forward HIV transmission, and comparison of various HIV programs. Our study is strengthened by the use of data from the largest integrated health care system in the U.S. and its national scope.

Studies assessing the comparative value of HIV retention measures are limited. Mugavero et al. recently evaluated commonly used retention measures in predicting viral load suppression at six academic HIV clinics [22]. Retention measures included count and dichotomous missed visits, visit adherence, 6-month gap, 4-month visit constancy, and the HRSA HAB retention measure. Among 10,053 patients included in the study, all six retention measures were significantly associated with viral load suppression at 12 months follow-up; however, there was variation in the correlation of each measure with viral load suppression. The authors suggested that their findings indicate no clear gold standard for measuring retention. Our study similarly found that all retention measures assessed were associated with virological suppression; however, we further assessed several other important clinical outcomes. Our data suggest that Missed Appointments might be the best measure of retention in HIV expert care.

Another recent study, by Yehia et al. sought to identify measures associated with retention in HIV expert care [38]. This was a large, multicenter, prospective cohort study of more than 17,000 HIV infected adults in the U.S. Three retention measures were assessed: gaps in care of >6 months between successive outpatient visits, quarterly visits, and having ≥2 visits at least 90 days apart annually. Each of these three measures correlated with retention in expert care (correlation coefficient ranged from 0.67 to 0.88). Additionally, all measures demonstrated similar patterns of associations with baseline characteristics (demographics and clinical variables). Although this study suggests that each of the three measures produces approximately similar estimates of HIV retention in expert care, the investigators did not evaluate clinical outcomes associated with each measure. Our study took the next step in assessing which retention measures are most likely to predict clinical outcomes. We also analyzed missed appointments, which could not be captured with the prior study design.

In our study, 36% of HIV patients missed appointments in ID clinics and 65% missed appointments in any clinic. Prior HIV studies have produced similar estimates of missed appointments (34 to 60%) [8,9]. Furthermore, our study suggests that missed appointments might be the most useful retention measure for predicting clinical outcomes. Patients might miss appointments if they feel poorly (worsening health) or are unmotivated to keep up with HIV-related care, both of which suggest worse clinical status [39]. Alternatively, patients who experience symptom improvement following initiation of ART might also miss appointments [40].

This association between missed appointments and clinical outcomes in HIV-infected patients has been demonstrated previously. Berg et al. conducted a retrospective cohort analysis in 995 HIV-infected patients at a single urban community health center [4]. The study examined the association of appointments missed in the prior year with suppression of HIV viral load and progression to AIDS. In this study, the number of missed appointments significantly predicted a detectable HIV viral load and an AIDS-defining CD4 cell count. In another study, Mugavero et al. conducted a prospective analysis of HIV patients receiving care at two outpatient university HIV treatment centers [8]. The primary outcome of this study was HIV viral load suppression. Among 676 patients evaluated, each missed appointment significantly prolonged the time to HIV viral load suppression. Conversely, visit adherence also significantly predicted lower cumulative HIV viral load. Finally, Lucas et al. conducted a retrospective analysis of 273 HIV-infected patients at a single HIV clinic to determine factors associated with failure to achieve virologic suppression [7]. Higher rates of missed clinic appointments were associated with virologic suppression at one year.

The results of these studies are in line with our own findings—missed appointments result in poorer clinical outcomes in HIV patients. These studies differed from ours in that they all evaluated only one measure of retention in care and they only included a limited number of outcome variables. Furthermore, some of these studies were limited in size and did not include patients from a nationally representative sample [7]. Finally, data collection was often limited to one year of follow-up; whereas our study followed patients for two years [4,7]. Despite these limitations, these studies strengthen the evidence that missed appointments are important predictors of clinical outcomes in HIV patients in high-income countries like the U.S.

Other measures of retention in HIV expert care have been shown to correlate with clinical outcomes. Giordano et al. demonstrated that patients who completed four appointments (at least one per quarter) within the year had significantly lower mortality compared to those with appointments in only one of the four quarters [5]. Additionally, patients who completed three or four appointments within the year had a significantly greater improvement in CD4 cell count and reduction in HIV viral load from baseline as compared to those with only one or two appointments within the year.

The differences in clinical outcomes among HIV patients who met the retention measures versus those who did not might be due to several factors. First, HIV-infected patients who miss clinic appointments might be poorly adherent to antiretroviral medications. This is problematic because patients who are poorly adherent to ART are more likely to develop resistance to ART [41]. In addition, our study found that patients who missed appointments or who had infrequent appointments were less likely to have initiated ART during 24 months of follow-up. Giordano et al. also demonstrated a significant association between missing two or more physician appointments and failure to receive ART [42]. Second, most HIV patients also need treatment for other non-HIV related chronic illnesses; thus, patients who miss physician appointments might also have poorly managed comorbid conditions [43]. Finally, missed appointments might limit the opportunity for patients to receive proper prophylactic therapy for other HIV-related illnesses. Each of these factors could lead to poorer clinical outcomes and increased resource utilization. Indeed, our study demonstrated increased resource utilization, including ED visits and hospitalizations, in patients who missed appointments.

Programs aimed at preventing missed appointments might be especially important given the results of our study. Practices could implement policies to track “no show” rates. They could then direct retention efforts to these patients to prevent no shows, such as a follow-up call after a missed appointment to express concern for the patient’s well-being and encourage them to come into the clinic. This approach has been useful in patients at high risk for suicide [44]. Other interventions should target factors commonly associated with patient adherence: health literacy, education, interpersonal relationships, patient-provider relationships, location of care, patient attitudes, and cultural variations [45]. Additionally, future research should focus on the development and testing of strategies to retain patients in care to maximize health outcomes.

Retaining HIV patients in expert care should continue to be a major priority for providers and public health organizations. Our findings suggest that the definition chosen to measure retention in HIV expert care might have implications for patient health outcomes. Missed appointments has been one of the most widely used retention measures in the literature [4,8,9]; however, variation in retention assessment can greatly hinder comparison of the effectiveness of various HIV programs [16]. Incorporating missed appointments into the NCQA, NQF, or HRSA HAB measure might aid in improving consistency of measures when evaluating retention across studies and settings and, ultimately, improving health outcomes among patients with HIV. Development of standardized national quality measures for HIV retention in care might also streamline care across the health care system and aid in reimbursement procedures for organizations who receive federal funding from the Centers for Medicare and Medicaid Services or HRSA.

This study has limitations. We studied a VHA population; therefore, study findings might not be generalizable to non-VHA care settings. VHA patients are mostly male, more likely to be African-American, and generally sicker than patients in other health care systems [46]. In addition, electronic medical data are created for the purpose of patient care, not for research, and might contain errors. There might be variation in the extent of physician reporting of patients’ medical conditions and this variability might lead to inaccuracy of the Charlson comorbidity score as well as other variables. We were also unable to determine patients’ prior HIV status, disclosure of status, care experiences prior to study inclusion, socioeconomic status, drug or substance abuse, or clinical severity. Furthermore, few (14%) patients in this study were married, suggesting possibly low social support. Each of these factors could have affected retention in care and possibly biased the retention measure-outcome relationship. Finally, the inclusion criteria for 24 month continuous care might bias this sample toward a better retained population or a higher population of early linkers. This could limit the generalizability of our study findings to other populations.

In conclusion, while all four measures of retention in HIV expert care had clinical value, Missed Appointments was the only measure that significantly predicted all primary and secondary outcomes at 24 months among VHA patients in HIV expert care. This finding could be useful to health care providers and public health organizations as they seek ways to optimize the health of HIV patients. Further studies are needed to confirm the value of measuring Missed Appointments in other HIV populations.
